# Managing Tumor Lysis Syndrome in the Era of Novel Cancer Therapies

**Published:** 2017-11-01

**Authors:** Ali McBride, Steven Trifilio, Nadine Baxter, Tara K. Gregory, Scott C. Howard

**Affiliations:** 1 The University of Arizona Cancer Center, Tucson, Arizona;; 2 Northwestern Memorial Hospital, Chicago, Illinois;; 3 University of Arkansas for Medical Sciences, Myeloma Institute for Research and Therapy, Little Rock, Arkansas;; 4 Colorado Blood Cancer Institute at Presbyterian St. Luke’s Medical Center, Denver, Colorado;; 5 University of Memphis, School of Health Studies, Memphis, Tennessee

## Abstract

**Abstract **

Tumor lysis syndrome (TLS) is a potentially life-threatening emergency that can develop rapidly after the release of intracellular contents from lysed malignant cells. The advent of novel and targeted therapies that have improved tumor-killing efficacy has the potential to increase the risk of TLS when used as part of front-line therapy. A recent review of TLS risk in patients with hematologic malignancies treated with newer targeted agents highlighted the need to revisit TLS risk stratification and to describe the practical challenges of TLS prevention, treatment, and monitoring. Although this era of rapid development of novel cancer therapies provides new hope for patients with hematologic malignancies, it is essential to be prepared for TLS because monitoring and prophylaxis can almost always prevent severe and life-threatening consequences. Heightened awareness of the development of TLS with novel and targeted agents, accompanied by aggressive hydration and rational, risk-appropriate management, are the keys to successful outcomes.

Tumor lysis syndrome (TLS) is a potentially life-threatening emergency that can develop rapidly after the release of intracellular contents from lysed malignant cells (Cairo, Coiffier, Reiter, Younes, & TLS Expert Panel, 2010; Howard, Jones, & Pui, 2011; Wilson & Berns, 2014). Characterized by hyperuricemia, hyperkalemia, hyperphosphatemia, and hypocalcemia, TLS results from the inability of homeostatic mechanisms to respond to the rapid release of intracellular contents such as nucleic acids (which are rapidly converted to uric acid), phosphate, and potassium into the blood (Howard et al., 2011; Wilson & Berns, 2014). It can occur spontaneously, especially in patients with high-grade malignancies, but it most commonly occurs soon after initiation of chemotherapy. Tumor lysis syndrome can lead to renal failure, arrhythmia, seizures, or death. Patients with less severe complications can suffer significant morbidity and increased health-care costs (Cairo et al., 2010; Mughal, Ejaz, Foringer, & Coiffier, 2010). Prompt management of TLS can reduce morbidity and mortality in patients being treated for hematologic malignancies, and identification of at-risk patients can often prevent TLS altogether. 

Cancer treatments have evolved from traditional cytotoxic chemotherapies to more targeted molecular or biologic agents with markedly different mechanisms of action. These novel targeted cancer agents have not only enhanced antitumor efficacy but also have fewer side effects and, in some cases, increased convenience when available as oral formulations. For example, in 2001, the tyrosine kinase inhibitor (TKI) imatinib was approved for the treatment of patients with chronic-phase chronic myeloid leukemia (CML) on the basis of unprecedented single-agent response rates, even among patients with interferon-resistant disease (O’Brien et al., 2003). Since then, enhanced understanding of the molecular aberrations underlying malignancy has enabled development of multiple targeted agents, including marketed drugs and many others in active clinical development. Despite their advantages and, indeed, because of their enhanced anticancer activity, such therapies may increase TLS risk, including in patients with diseases not previously linked to TLS (e.g., chronic lymphocytic leukemia [CLL] and multiple myeloma). However, the extent to which these novel targeted agents influence the risk of TLS in a given tumor type is not well characterized. 

In this article, we provide a practical guide on TLS in the context of new cancer therapies; review previously published strategies for identifying TLS risk; discuss key insights from a systematic literature review (Howard, Trifilio, Gregory, Baxter, & McBride, 2015); revisit risk stratification in light of new therapies; and highlight practical challenges of TLS prevention, treatment, and monitoring, with insights applicable to community practice. 

## CURRENT MODELS TO IDENTIFY PATIENTS AT RISK FOR TLS

Current schemas for identifying TLS risk factors focus primarily on tumor and patient characteristics (Cairo et al., 2010; Howard et al., 2011; Mughal et al., 2010). In 2010, an expert panel published recommendations for evaluating TLS risk (low, intermediate, or high) in adult and pediatric patients with cancer. Risk was classified by the type of malignancy, with solid tumors generally considered low risk, except for bulky chemotherapy-sensitive entities such as neuroblastoma, germ cell tumors, and small cell lung cancer, which were categorized as intermediate risk (Cairo et al., 2010). Hematologic malignancies were also first characterized by type, with multiple myeloma and the chronic leukemias grouped under the low-risk category, except for CLL treated with targeted and/or biologic therapies, which increased the risk for TLS to intermediate (CLL treated with alkylating agents was low risk). Acute leukemias were further stratified as acute myeloid leukemia (AML), acute lymphoblastic leukemia (ALL), or Burkitt lymphoma/leukemia. All Burkitt leukemias were considered high risk, as were AML or ALL with white blood cell (WBC) counts ≥ 100 × 10^9^/L and ALL with a WBC count < 100 × 10^9^/L but lactate dehydrogenase ≥ 2 × the upper limit of normal. Classification of TLS risk with various lymphomas followed an increasingly complex algorithm.

For all malignancies, risk was adjusted at the final step based on renal function, with shifts to higher-risk categories for patients with low- or intermediate-risk and renal dysfunction and/or renal involvement or for those with normal renal function but elevated uric acid, phosphate, or potassium levels (Cairo et al., 2010). With only one exception (increased TLS risk in patients with CLL who receive biologic or targeted treatment), the type of therapy was not used to determine TLS risk.

**Figure 1:**
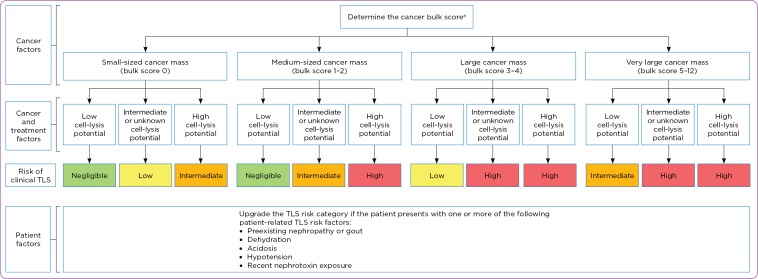
Tumor lysis syndrome risk stratification based on cancer, treatment, and patient factors. TLS = tumor lysis syndrome. Used with kind permission of Scott Howard. (See Table 1).

A second publication considered TLS risk based on four major categories of risk factors: cancer mass, cell-lysis potential of the tumor, patient characteristics at presentation, and supportive care (Howard et al., 2011). The potential for cell lysis depends not only on the chemosensitivity of the cancer cells but also on the intensity (or efficacy) of the initial anticancer therapy used, with higher intensity (efficacy) conferring a higher risk. Given the potential of highly effective novel and targeted therapies to increase the risk of TLS when used as part of initial anticancer therapy, we recommend that the particular type of therapy receive increased focus when assessing risk. Table 1 provides a practical guide for assessing tumor bulk in hematologic malignancies and can be utilized in an algorithm for TLS risk stratification based on cancer, cancer treatment, and patient factors (Figure 1).

**Table 1:**
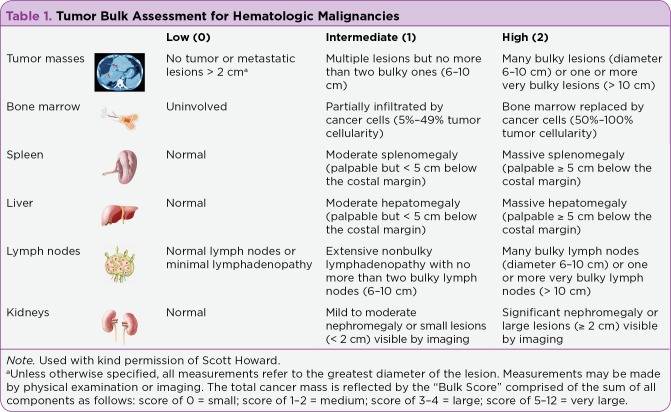
Tumor Bulk Assessment for Hematologic Malignancies

## RISK FOR TLS WITH NEW AND EMERGING TREATMENTS

To explore whether new and emerging agents are associated with TLS, we conducted a systematic review of TLS associated with selected molecular and biologic agents. Articles published between January 2010 and October 2014 for phase I to III clinical trials in hematologic malignancies with novel monoclonal antibodies, TKIs, protease inhibitors, chimeric antigen receptor (CAR) T cells, and the proapoptotic agent lenalidomide (Revlimid) were retrieved from the literature and analyzed (Table 2; Howard et al., 2015).

**Table 2:**
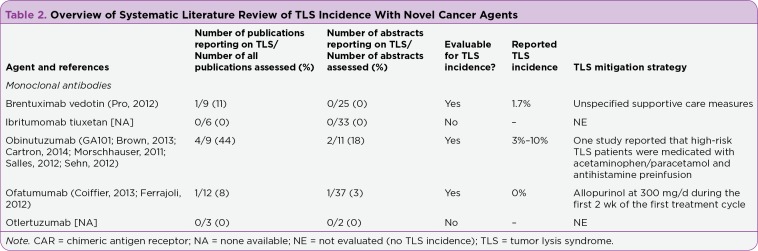
Overview of Systematic Literature Review of TLS Incidence With Novel Cancer Agents

**Table 2 (cont.):**
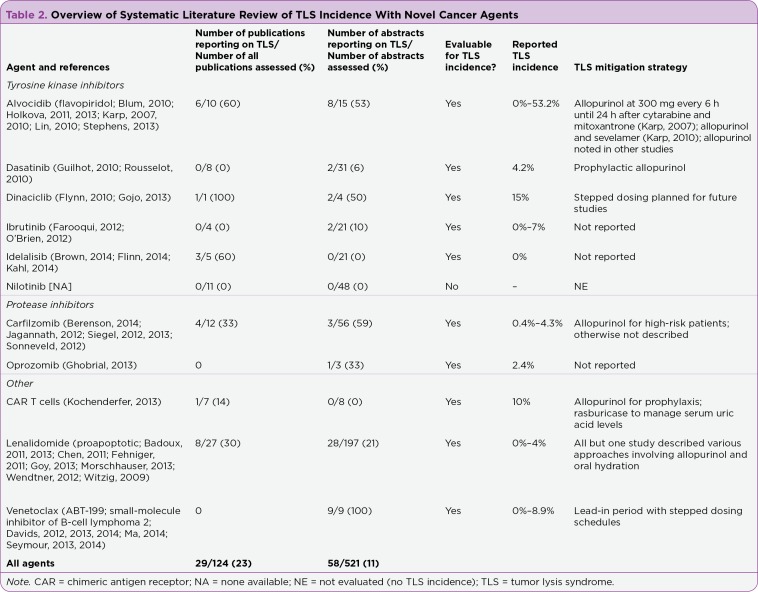
Overview of Systematic Literature Review of TLS Incidence With Novel Cancer Agents (cont.)

Overall, the incidence of TLS varied greatly across agents and cancer types. With the use of two agents, idelalisib (Zydelig) and ofatumumab (Arzerra), no TLS was reported. Agents for which the TLS incidence was < 5% included brentuximab vedotin (Adcetris; Pro et al., 2012), carfilzomib (Kyprolis; Berenson et al., 2014; Jagannath et al., 2012; Siegel et al., 2013; Siegel et al., 2012; Sonneveld et al., 2012), dasatinib (Sprycel; Rousselot et al., 2010), lenalidomide (Badoux et al., 2011; Badoux et al., 2013; Chen et al., 2011; Fehniger et al., 2011; Goy et al., 2013; Morschhauser et al., 2013; Wendtner et al., 2012; Witzig et al., 2009), and oprozomib (Ghobrial et al., 2013).

Two phase I trials of venetoclax (ABT-199; Venclexta) reported a TLS incidences > 5% (Seymour et al., 2013, 2014) in relapsed or refractory CLL (8.3% and 8.9%, respectively), with one fatality per trial. These fatalities led to interruption of the clinical development of ABT-199 and resulted in a stepwise dosing schema specifically to mitigate TLS risk. The incidence of TLS was 10% in a 10-patient phase II trial of CAR T cells (Kochenderfer et al., 2013) and in a 40-patient phase II trial of obinutuzumab in relapsed or refractory aggressive non-Hodgkin lymphoma (NHL; Morschhauser et al., 2011). A phase II trial of dinaciclib in 20 patients with advanced ALL or AML reported a 15% TLS incidence, including 1 fatality from acute renal failure despite aggressive prophylaxis and hemodialysis (in a patient with AML and pretreatment laboratory TLS; Gojo et al., 2013). The highest incidences of TLS, 42% and 53%, occurred in phase II studies of alvocidib in a sequential regimen with cytarabine and mitoxantrone in poor-risk AML (Karp et al., 2007, 2010). 

## CLINICAL CHALLENGES

**TLS Management and Control of Uric Acid**

Prophylactic management for patients at risk for TLS consists of vigorous hydration, therapies that decrease the production of uric acid (e.g., allopurinol) or enzymatically remove it (e.g., rasburicase [Elitek]; Howard et al., 2011; Wilson & Berns, 2014) as well as the avoidance of exogenous potassium and phosphorus. Because the primary goal of prophylactic management is prevention of clinical TLS, close monitoring is required to detect metabolic abnormalities before they cause symptoms.

The current treatment options for controlling uric acid in the setting of TLS are allopurinol and rasburicase. Allopurinol reduces the production of uric acid but has no effect on current uric acid levels. Rasburicase can immediately reduce existing uric acid levels and is also effective when the tumor burden is anticipated to be high, thus alleviating the risk of tumor lysis. Recently, febuxostat (Uloric), a non–purine selective inhibitor, was approved by the US Food and Drug Administration (FDA) for chronic management of hyperuricemia in patients with gout (Takeda Pharmaceuticals U.S.A., Inc., 2013), is currently being studied in the TLS setting.

**Figure 2:**
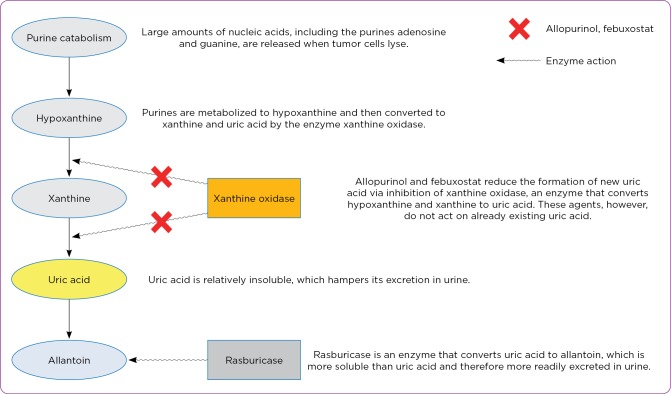
Mechanism of action for allopurinol, febuxostat, and rasburicase. Information from Sanofiaventis (2011); Hu & Tomlinson (2008); Pession et al. (2008).

Allopurinol and febuxostat inhibit xanthine oxidase, the enzyme that converts hypoxanthine to xanthine and xanthine to uric acid (Figure 2; Hu & Tomlinson, 2008; Pession, Melchionda, & Castellini, 2008), and thus prevent the formation of new uric acid from purines released by cancer cell lysis, reducing both serum and urinary levels of uric acid. In a phase III clinical trial, allopurinol (300 mg/day orally) administered to adults on days 1 to 5 resulted in a response rate (defined as uric acid levels ≤ 7.5 mg/dL for all measurements from days 3–7) of 66% (95% confidence interval [CI] = 56%–76%; Cortes et al., 2010). When used to prevent uric acid nephropathy from anticancer therapy, it is recommended to administer as a high volume of fluid with allopurinol 600 to 800 mg/day orally (Prometheus Laboratories Inc., 2009). The allopurinol dose must be adjusted, however, in patients with acute kidney injury because it is renally cleared. A dose-dependent decrease in serum and urinary uric acid levels can be expected within 2 to 3 days.

Skin rashes or other signs or symptoms of an allergic reaction should prompt immediate discontinuation, with further use contraindicated after any severe reactions (allergic or otherwise). Bendamustine carries a warning against the concomitant use of allopurinol because of severe skin reactions (such as Stevens-Johnson syndrome; Teva Pharmaceutical Industries Ltd., 2016). Recently, febuxostat was shown to reduce elevated uric acid levels associated with TLS, with efficacy comparable (Maie et al., 2014) or superior to allopurinol (Spina et al., 2015).

Rasburicase is a recombinant urate oxidase that reduces uric acid blood levels by converting uric acid into allantoin (Figure 2), which is more soluble than uric acid (Bose & Qubaiah, 2011). Rasburicase is indicated for the initial management of plasma uric acid levels in patients (pediatric and adult) with leukemia, lymphoma, and solid tumors in whom chemotherapy would be expected to result in TLS and plasma uric acid elevation (Howard et al., 2011; Sanofi-aventis, 2011). The FDA-approved regimen for rasburicase is 0.2 mg/kg/day as a 30-minute intravenous infusion for up to 5 days (Sanofi-aventis, 2011).

Eight studies supported regulatory approval, including studies demonstrating significantly greater uric acid lowering vs. allopurinol in pediatric acute leukemia or NHL (Goldman et al., 2001) and higher response rates vs. allopurinol in adult leukemia, lymphoma, or other hematologic malignancy (87% vs. 66%; Cortes et al., 2010). After considering accumulating data from compassionate-use trials in which rasburicase demonstrated efficacy at lower doses and over a shorter duration of therapy, an expert panel concluded that dosing may be 0.1, 0.15, and 0.2 mg/kg for low-, intermediate-, and high-risk disease, respectively, for 1 to 7 days (Coiffier, Altman, Pui, Younes, & Cairo, 2008). Note, however, that although not evidence based, fixed-dose regimens are used in some institutions.

One retrospective series used fixed low doses of rasburicase of 1.5, 3, 4.5, or 6 mg to treat hyperuricemia secondary to TLS, with a response rate of 80% (reduction of uric acid to < 8 mg/dL in 31 of 39 adult patients with a baseline above this threshold) after initial dosing (Herrington & Dinh, 2014). In the largest retrospective study (N = 373) conducted in patients with hematologic malignancy or solid tumors who were receiving rasburicase for TLS prevention (McBride et al., 2013), no difference was observed for the achievement of normalized plasma uric acid level (< 7.5 mg/dL) 24 hours after administration of a single fixed (3, 6, or 7.5 mg) or weight-based (≥ 0.1 mg/kg) rasburicase dose. Other studies of fixed-dose rasburicase have demonstrated uric acid–lowering efficacy (Herrington & Dinh, 2014). Unfortunately, no adequately powered study of low-dose vs. fixed-dose vs. standard-dose rasburicase has been conducted in patients at intermediate risk of TLS, so optimal use of rasburicase in this group remains controversial.

Rasburicase is contraindicated in patients with a history of anaphylaxis or severe hypersensitivity reactions to rasburicase. Repeated exposure to rasburicase can lead to hypersensitivity reactions in patients who receive an initial course of rasburicase and later require another dose (or series of doses) to manage TLS at the time of relapse (Allen et al., 2015). Rasburicase is also contraindicated in patients with glucose-6-phosphate dehydrogenase (G6PD) deficiency, an X-linked condition associated with a risk for hemolytic anemia and potential methemoglobinemia consequent to the hydrogen peroxide production that occurs with rasburicase (Relling et al., 2014; Sanofi-aventis, 2011). In clinical studies, hemolysis or methemoglobinemia occurred in < 1% of patients who received rasburicase (Sanofi-aventis, 2011).

Patients with G6PD deficiency should receive allopurinol instead of rasburicase (Cairo et al., 2010). Men of African, Middle Eastern, or Mediterranean origin have an incidence of G6PD deficiency as high as 20% (Relling et al., 2014), although it should be noted that patients can carry a G6PD deficiency irrespective of ancestry. Optimally, testing would occur at the time of intake into the oncology practice before rasburicase administration (Relling et al., 2014). Established deficiency per genotyping is sufficient for contraindication, but the limitations of genotyping typically warrant G6PD enzyme testing.

If uric acid levels are high and testing will be delayed, patients with no personal/family history suggestive of G6PD deficiency may receive a small dose of rasburicase (1.5 mg), with testing 4 hours later for laboratory evidence of hemolysis (i.e., changes in hemoglobin, haptoglobin, and bilirubin; Elinoff, Salit, & Ackerman, 2011). If uric acid levels remain high and no hemolysis is evident, additional rasburicase can be administered safely. For those with hematologic cancers, G6PD activity should be measured at presentation because many patients receive red blood cell transfusions soon after diagnosis, and transfusion can mask G6PD deficiency (Bucklin & Groth, 2013). 

**Use of Uric Acid–Lowering Medications Based on TLS Risk**

Allopurinol is typically used in patients at low risk of clinical TLS, and rasburicase is used in those at high risk (or who present with TLS). For intermediate-risk patients, a single dose of rasburicase may be used to eliminate existing uric acid, followed by allopurinol to prevent formation of new uric acid. Alternatively, initial allopurinol use (to decrease new uric acid formation) may be combined with hyperhydration to remove existing uric acid. Regardless of the initial chosen strategy, serial uric acid measurements should guide continued therapy. If uric acid increases despite allopurinol, rasburicase can be used as subsequent rescue; however, this strategy has the disadvantage of allowing accumulation of xanthine, which is not removed by subsequent rasburicase administration.

For individualized selection of rasburicase dose, considerations include the patient’s baseline uric acid level, rate of increase from the baseline uric acid level, anticipated increase due to tumor load, effectiveness of therapy to lyse the tumor, and the uric acid goal. It is important to note that repeat dosing may be necessary for patients in whom initial dosing does not achieve the desired reduction in uric acid levels after 4 hours (Coiffier et al., 2008), with the potential to eliminate the need for hemodialysis in these patients who may have highly chemosensitive or rapidly dividing tumors. In our experience, a single dose of rasburicase may also be used in patients at intermediate risk of clinical TLS, with reassessment to determine the need for a second dose (Howard et al., 2011). Regardless of the initial TLS risk category or initial therapy, patients who develop acute kidney injury and have persistent hyperuricemia while taking allopurinol should receive a dose of rasburicase to remove existing uric acid and mitigate the risk for ongoing renal damage.

Obese patients are of particular concern for uric acid management. In a single-center retrospective analysis of 151 patients, body mass index did not correlate with failure of fixed-dose rasburicase in adults, but the study did not have sufficient statistical power to compare outcomes in obese vs. nonobese patients, particularly in the relevant (controversial) subset at intermediate risk for TLS (Clemmons, Ensley, Hoge, & Clark, 2014). The study’s findings were similar to those published in a previous report (McBride et al., 2013). 

Although earlier consensus guidelines prohibited the concomitant use of allopurinol with rasburicase to avoid xanthine accumulation and lack of substrate for rasburicase (Tosi et al., 2008), limited evidence suggests this combination may be beneficial, although not synergistic, and in general rasburicase is so effective at reducing uric acid that once a decision is made to administer it, allopurinol would not be necessary as an adjunct (Cortes et al., 2010). With either allopurinol or rasburicase, avoiding concomitant drugs that increase uric acid (Table 3) is recommended. 

**Table 3:**
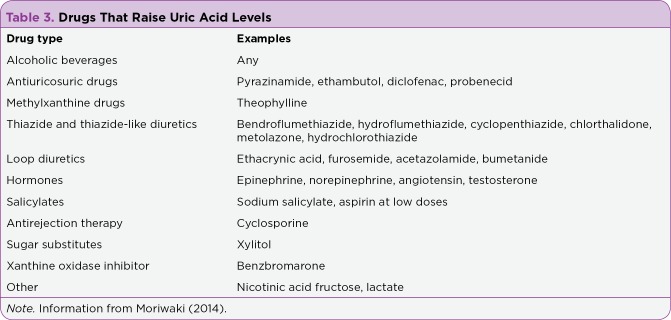
Drugs That Raise Uric Acid Levels

The National Comprehensive Cancer Network (NCCN) recommendations include both allopurinol and rasburicase as options, specifying that rasburicase is appropriate for NHL patients with certain risk factors (i.e., high-risk features, high-bulk disease, inadequate hydration, acute renal failure; NCCN, 2014a) and should be considered as initial treatment in AML associated with rapidly increasing blasts, high uric acid, or impaired renal function (NCCN, 2014b). The NCCN NHL Guidelines note that allopurinol should begin 2 to 3 days before chemotherapy and continue for 10 to 14 days, whereas one dose of rasburicase is frequently adequate (NCCN, 2014a). Intravenous rather than oral allopurinol can be used; however, 2 to 3 days are still required to reduce uric acid levels, because the mechanism of action is not the removal of existing uric acid but a reduction in the formation of new uric acid.

**Monitoring in Emergent and Community Settings**

Published guidelines suggest that laboratory and clinical TLS parameters (levels of uric acid, phosphate, potassium, calcium, and lactate dehydrogenase in addition to measurements of fluid input and urine output) should be monitored 4 to 6 hours after initial chemotherapy administration in pediatric patients at high risk (Coiffier et al., 2008; Howard et al., 2011). These guidelines recommend that adult patients at intermediate risk be monitored for at least 24 hours after the completion of chemotherapy (Coiffier et al., 2008). Our recommendation is to tailor the monitoring interval according to the three categories of TLS risk (Table 4), with even more intense monitoring in the event of treatment-emergent clinical TLS (Howard et al., 2011). 

**Table 4:**
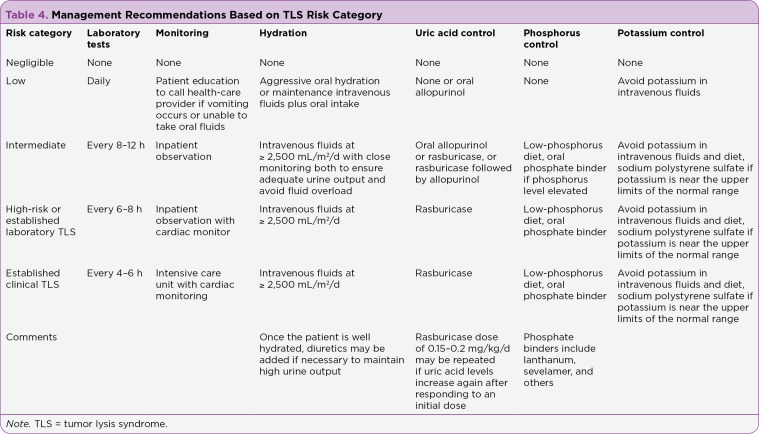
Management Recommendations Based on TLS Risk Category

The optimal frequency of electrolyte and uric acid monitoring depends on the clinical TLS risk. In patients who receive rasburicase, monitoring should occur every 4 to 6 hours to ensure that serum uric acid is within a safe range. We give additional rasburicase doses (at a reduced dose) as needed whenever uric acid starts to increase again after a previous dose; and we continue monitoring until clinical laboratory parameters (including a complete metabolic panel, complete blood cell count, magnesium, phosphorus, as well as uric acid) are within normal limits or until chemotherapy induction is completed. However, if low doses of rasburicase (< 0.15–0.2 mg/kg examined in prospective clinical trials) are used initially, uric acid may require more frequent monitoring to detect rising levels. 

It is important to note that rasburicase results in enzymatic degradation of uric acid ex vivo when blood samples are drawn and remain at room temperature. To avoid artificially low measured uric acid levels in patients who have received rasburicase, blood samples should be collected in prechilled heparin-containing tubes and immediately transferred to an ice water bath, with centrifugation utilizing a precooled centrifuge. At that time, plans need to be in place to assay the samples within 4 hours after collection. 

**Management Practices With New Agents for Hematologic Cancers**

Our survey of TLS in association with new agents yielded little specific information on the mitigation strategies employed. For alvocidib, mitigation utilized oral allopurinol at 300 mg every 6 hours until 24 hours after cytarabine and mitoxantrone in the initial trial (Karp et al., 2007), with both allopurinol and sevelamer in the subsequent trial (Karp et al., 2010). Mitigation of TLS was employed across all of the clinical trials of alvocidib that reported TLS (either specifying allopurinol or stated more generally and additionally delaying alvocidib initiation in one combination trial). Routine TLS prophylaxis occurred in clinical trials of lenalidomide due to a high rate seen in an early clinical trial in CLL (Moutouh-de Parseval, Weiss, DeLap, Knight, & Zeldis, 2007). Including fatalities, TLS occurred during the early clinical development of ABT-199, prompting the protocols to adopt a stepwise dosing schema specifically intended to mitigate TLS risk. Rasburicase was rarely mentioned, except in a phase I trial of alvocidib in combination with cyclophosphamide and rituximab in high-risk CLL in which it was given with the first two courses as part of a comprehensive regimen that also included allopurinol at 300 mg, hydration, urine alkalinization, and a phosphate binder (Stephens et al., 2013). Overall, the TLS mitigation strategy was not mentioned or was stated in general terms for a number of trials (Table 2), questioning whether the protocols had captured mitigation in their supportive care guidelines. 

## KNOWLEDGE GAPS

Knowledge gaps are as follows:

- Information on the specific TLS prophylaxis strategies that are used in clinical trials of novel cancer agents for hematologic malignancies is rarely reported (Table 2).
- The limited available clinical trial evidence suggests that TLS is an important complication of novel and targeted therapies, which varies across agents and tumor types.
- Dinaciclib-treated patients with advanced leukemias, venetoclax-treated patients with CLL, and especially alvocidib-treated patients with AML appear to be at highest risk of TLS, although there is at least some risk with most surveyed agents (Table 1; Howard et al., 2015).
- As these molecules become more widely used, it is essential to learn more about which strategies should be initiated, with consideration of the type of therapy being a key factor in the overall risk assessment.

The overarching goal of TLS prevention is avoidance, yet breakthrough cases occur despite aggressive prophylaxis. For example, in the randomized trial of rasburicase alone, rasburicase plus allopurinol, or allopurinol alone in patients with hematologic malignancies at high risk for hyperuricemia and TLS, the incidences of clinical TLS (at least two laboratory abnormalities coupled with signs or symptoms of organ damage, including acute kidney injury, seizure, or arrhythmia) was 3%, 3%, and 4%, respectively, with corresponding laboratory TLS rates of 21%, 27%, and 41% and acute renal failure rates of 2%, 5%, and 2% (Cortes et al., 2010). All patients received an initial dose of study medication 4 to 24 hours before initiating chemotherapy. It was recommended, but not required, that normal or half-normal saline be initiated at a rate of 4 to 5 L/day, starting 24 to 48 hours before chemotherapy.

The extent to which different practices of hydration, left to investigator discretion, may have influenced the TLS rates is unknown. Elderly patients and those with impaired cardiac and renal function are often unable to tolerate vigorous hydration and may therefore be candidates for early rasburicase treatment. The safety of reduced doses of rasburicase requires a prospective study in patients at intermediate TLS risk. In addition, concomitant rasburicase for upfront prophylaxis require further evaluation based on current reported differences in dosing between these investigational agents, as seen with alvocidib and other agents.

Moving forward, community oncologists, nurses, advanced practitioners and others who care for patients with hematologic malignancies would benefit from a clear-cut pathway for determining the optimal approaches for prevention and management of TLS as they pertain to a given cancer agent. In the hope that our experience in treating patients with TLS may be useful to others, we offer a collection of clinical pearls (Table 5) and a suggested approach to patient management (Table 6).

**Table 5:**
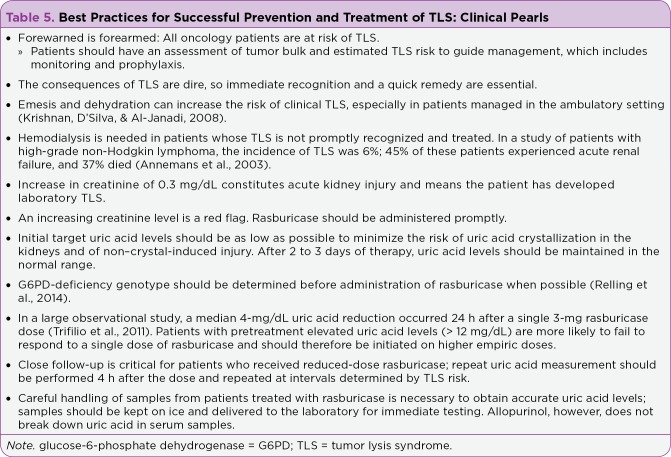
Best Practices for Successful Prevention and Treatment of TLS: Clinical Pearls

**Table 6:**
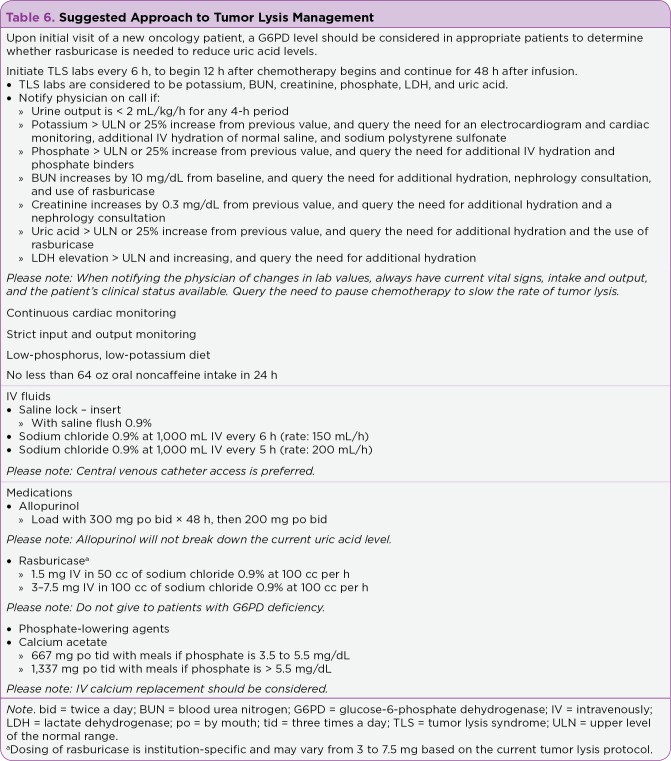
Suggested Approach to Tumor Lysis Management

## CONCLUSIONS

This era of rapid development of novel cancer therapies provides new hope for patients who suffer from hematologic malignancies. However, the promise of these agents can only be realized by avoiding death or complications from TLS, which is due, paradoxically, to the very nature of their higher efficacy. Heightened awareness of the development of TLS with novel and targeted agents, accompanied by aggressive hydration and rational therapy, is key to successful treatment outcomes.

**Acknowledgments**

Editorial and medical writing support was provided by Laurie Orloski, PharmD, and Mariana Ovnic, PhD, of Complete Publication Solutions, LLC. This support was provided by Sanofi-aventis.
